# First-in-man tau vaccine targeting structural determinants essential for pathological tau–tau interaction reduces tau oligomerisation and neurofibrillary degeneration in an Alzheimer’s disease model

**DOI:** 10.1186/alzrt278

**Published:** 2014-08-01

**Authors:** Eva Kontsekova, Norbert Zilka, Branislav Kovacech, Petr Novak, Michal Novak

**Affiliations:** 1 AXON Neuroscience, Dvorakovo nabrezie 10 811 02, Bratislava, Slovak Republic; 2 Present address: Institute of Neuroimmunology, Dubravska cesta 9, 84510 Bratislava, Slovak Republic

## Abstract

**Introduction:**

We have identified structural determinants on tau protein that are essential for pathological tau–tau interaction in Alzheimer’s disease (AD). These regulatory domains, revealed by monoclonal antibody DC8E8, represent a novel target for tau-directed therapy. In order to validate this target, we have developed an active vaccine, AADvac1.

**Methods:**

A tau peptide encompassing the epitope revealed by DC8E8 was selected for the development of an active vaccine targeting structural determinants on mis-disordered tau protein that are essential for pathological tau–tau interaction. The efficacy of the vaccine was tested in a transgenic rat model of human tauopathies. Toxicology and safety pharmacology studies were conducted under good laboratory practice conditions in multiple rodent and nonrodent species.

**Results:**

We have administered the tau peptide vaccine to a rat model of AD to investigate whether the vaccine can improve its clinical, histopathological and biochemical AD phenotype. Our results show that vaccination induced a robust protective humoral immune response, with antibodies discriminating between pathological and physiological tau. Active immunotherapy reduced the levels of tau oligomers and the extent of neurofibrillary pathology in the brains of transgenic rats. Strikingly, immunotherapy has reduced AD-type hyperphosphorylation of tau by approximately 95%. Also, the tau peptide vaccine improved the clinical phenotype of transgenic animals. Toxicology and safety pharmacology studies showed an excellent safety and tolerability profile of the AADvac1 vaccine.

**Conclusions:**

Active immunisation targeting crucial domains of Alzheimer tau eliminated tau aggregation and neurofibrillary pathology. Most importantly, the AD type of tau hyperphosphorylation was abolished by vaccination across a wide range of AD phospho-epitopes. Our results demonstrate that active immunisation led to elimination of all major hallmarks of neurofibrillary pathology, which was reflected by a profound improvement in the clinical presentation of transgenic rats. This makes the investigated tau peptide vaccine a highly promising candidate therapeutic for the disease-modifying treatment of AD. The tested vaccine displayed a highly favourable safety profile in preclinical toxicity studies, which opens up the possibility of using it for AD prophylaxis in the future. The vaccine has already entered phase I clinical trial under the name AADvac1.

**Trial registration:**

Current Controlled Trials NCT01850238. Registered 7 May 2013.

## Introduction

Over the course of Alzheimer’s disease (AD), neurofibrillary pathology spreads through the human brain, gradually disabling affected regions and leading to a decline in cognitive function. The spatial distribution and severity of neurofibrillary lesions closely correlates with cognitive impairment and brain atrophy observed in AD [1-3]. With no effective therapy available and the continuing aging of the population, the number of patients is rising quickly. More than 30 million people in the world today are affected by dementia, and it is predicted that the number of patients will reach over 100 million by 2050 [4]. Current pharmacological treatment of AD is based on the use of acetylcholinesterase inhibitors that are able to produce moderate symptomatic benefits for over 12 months [5]. However, they could not halt disease progression. It is important to note that no new drug against AD has been marketed for almost 17 years. Therefore, there is a huge demand for the development of disease-modifying drugs for AD that could attenuate or even reverse the neurodegenerative process by targeting a major hallmark of the disease, such as neurofibrillary degeneration.

The concept of immunotherapy has gained a strong foothold in the AD field [6]. Up to now, several approaches to immunotherapy have been tested in clinical studies with the aim to counteract amyloid pathology and thus improve cognition [7]. Despite the fact that active and passive immunisation against amyloid-β (Aβ) has been shown to clear or prevent Aβ brain plaques and improve cognitive performance in numerous mouse model studies [8], large-scale trials of several immunotherapeutics targeting Aβ have displayed little or no cognitive efficacy [6]. Therefore, much attention is now directed to immunotherapy targeting tau protein [9-12]. Several independent studies have shown that active and passive immunisation approaches were effective in reducing the burden of neurofibrillary tangles (NFTs) in the brain, slowing the progression of the behavioural phenotype or delaying the onset of motor function decline and weight loss in mouse models of tau tangle pathology [13-19].

Currently proposed tau immunotherapeutic approaches are selectively targeting individual phosphorylated tau (phospho-tau) epitopes such as phospho-Ser396/phospho-Ser404 [13,14,16], phospho-Thr231/phospho-Ser235 [20] or phospho-Ser422 [19]. However, tau is a phosphoprotein that contains 85 potential serine, threonine and tyrosine phosphorylation sites. Mass spectrometric analysis, combined with sequencing achieved by Edman degradation and specific antibody reactivity, showed that almost 10 phosphorylation sites on tau can be detected in the normal brain (Ser46, Thr181, Ser199, Ser202, Thr231, Ser404, Ser412/Ser413/Thr414 and Ser416), and approximately 45 phosphorylation sites have been found in the AD brain [21,22]. These results suggest that some of the phospho-sites targeted by proposed immunotherapies are present in healthy human brains. This finding raises concerns about the safety of vaccines targeting specific phospho-tau species. On the other hand, these therapeutic approaches can selectively target only one or two phospho-sites from among those forty-five identified in the AD brain. Moreover, the functional significance of the targeted phospho-sites remains unclear. Likewise, it is unknown whether all of the pathologic AD phospho-epitopes coexist on the same tau molecule or whether distinct subspecies of pathological tau display a variety of different phospho-codes. On the basis of these limitations, one can assume that effective immunotherapy against diseased forms of tau should attack a common denominator of the diseased tau proteome—the vulnerable area on tau responsible for oligomerisation, independently from the variety of its phospho-patterns. Therefore, we have focused our immunotherapeutic approach on structural determinants essential for pathological tau–tau interactions with the intent to prevent tau hyperphosphorylation, oligomerisation and development of neurofibrillary degeneration in an AD rat model.

## Methods

### Monoclonal antibodies

AT8 (recognises pSer202/pThr205) was purchased from Innogenetics (Ghent, Belgium). The phosphorylation-specific monoclonal antibodies (mAbs) DC209 (recognises pThr231), DC217 (recognises pThr217) and DC179 (recognises pThr181) were prepared by AXON Neuroscience SE (Bratislava, Slovak Republic). As a pan-tau antibody, mAb DC25 was used (AXON Neuroscience SE) [23-29]. DC25 recognises a stretch of seven amino acid residues (347 to 353, the longest human tau isoform numbering) in the fourth microtubule-binding repeat of tau, which is common to all neuronal tau protein isoforms (human, mouse, rat and other) and recognises all forms of physiological and pathological tau proteins.

### Design and construction of the active vaccine

Tau peptide sequence ^294^KDNIKHVPGGGS^305^ was derived from the regulatory domain driving the oligomerisation of tau [30]. An extra N-terminal cysteine residue was added with the aim of obtaining oriented attachment of the peptide on the surface of the keyhole limpet haemocyanin (KLH) protein. The peptide was coupled to the KLH carrier (Calbiochem, San Diego, CA, USA) via the bifunctional cross-linker *N*-[γ-maleimidobutyryloxy]succinimide ester (GMBS). KLH (20 mg) was dissolved in conjugation buffer (phosphate-buffered saline (PBS) with 0.9 M NaCl) to a concentration of 10 mg/ml. GMBS (2 mg) was dissolved in 50 μl of anhydrous dimethylformamide and mixed with the KLH solution for 1 hour at room temperature (RT). Subsequently, unreacted GMBS was removed on a 5-ml HiTrap desalting column (GE Healthcare Bio-Sciences, Pittsburgh, PA, USA) equilibrated in the conjugation buffer. The peptide (20 mg) was dissolved in the maleimide-activated KLH solution and the reaction proceeded for 2 hours at RT. The resulting conjugate was dialysed against PBS. The conjugate was aliquoted and stored at 2°C to 8°C until use.

### Transgenic animals

Transgenic rats used in this study expressed human truncated tau protein in the brain and spinal cord [25]. All transgenic rats used in this study were hemizygous for the transgene construct. Prior to the experiments, all animals were housed under standard laboratory conditions with free access to water and food and were kept under diurnal lighting conditions (12-hour light–dark cycles with light starting at 7:00 AM). All experiments on animals were carried out according to institutional animal care guidelines conforming to international standards and were approved by the State Veterinary and Food Committee of the Slovak Republic (Ro-2426/08-221) and by the Ethics Committee of the Institute of Neuroimmunology (15 October 2008), Slovak Academy of Sciences, Bratislava. Efforts were made to minimise the number of animals utilised.

### Vaccine administration

To prepare the tau peptide vaccine, 100 μg of tau peptide conjugate (dissolved in 150 μl of PBS) was mixed at a 1:1 (vol/vol) ratio with Adju-Phos adjuvant (Brenntag Biosector, Frederikssund, Denmark) in a final dose volume of 300 μl. The suspension was mixed by end-over-end rotation during incubation at 4°C for 6 hours to allow the peptide conjugate to adsorb onto the adjuvant. The transgenic rats received five subcutaneous injections of vaccine (100 μg of peptide-KLH conjugate/dose) starting at 2 months of age, followed by the second injection 3 weeks later and thereafter on a monthly schedule. The control group of transgenic rats received adjuvant mixed 1:1 with PBS in a final dose volume of 300 μl. Sera were collected 2 weeks after the last booster dose.

### Determination of antibody response to vaccine

Tau peptide (294 to 305), mis-disordered tau (151-391/4R) and recombinant full-length tau isoform 2N4R were separately coated onto 96-well plates (SARSTEDT, Nümbrecht, Germany) at an amount of 250 ng/well. After blocking, the plates were incubated with the serial dilutions of the sera (50 μl/well 1:100 to 1:51,200 in PBS, in twofold dilution steps) for 1 hour at 37°C. Bound serum antibodies were detected with peroxidase-conjugated secondary antibody (goat anti-rat immunoglobulin (Ig), Dako, Glostrup, Denmark) using chromogenic substrate *o*-phenylenediamine (Sigma-Aldrich, St Louis, MO, USA).

### Determination of isotypic profile of vaccine-induced antibodies

To determine the isotypes of the specific antibodies produced in response to vaccine, sera from immunised rats were serially diluted from 1:100 to 1:12,800 in twofold dilution steps and tested in duplicates by enzyme-linked immunosorbent assay (ELISA) against mis-disordered tau (151-391/4R). To detect rat IgG1, IgG2a, IgG2b, IgG2c and IgM isotypes, anti-rat subclass-specific horseradish peroxidase (HRP)–conjugated secondary antibodies were diluted 1:5,000 in PBS (Pierce Biotechnology, Rockford, IL, USA). Antibody isotype levels were compared based on the half-maximal effective concentration value of the dilution factor.

### Isolation of soluble tau and sarkosyl-insoluble tau

Brain tissue was homogenised in a tenfold weight excess of ice-cold extraction buffer (20 mM Tris, pH 7.4, 800 mM NaCl, 1 mM ethylene glycol tetraacetic acid), 1 mM ethylenediaminetetraacetic acid, 0.5% β-mercaptoethanol, 10% sucrose and 1 mM Na_3_VO_4_; 20 mM NaF, supplemented with cOmplete Protease Inhibitor Cocktail Tablet (Roche Diagnostics, Indianapolis, IN, USA). After incubation on ice for 5 minutes, the homogenates were cleared by centrifugation at 20,000 *g* for 20 minutes at 4°C. The supernatants were collected, and the total protein concentration was determined using the Bio-Rad protein assay (Bio-Rad Laboratories, Hercules, CA, USA). This supernatant (designated 1S) contained soluble tau fraction. Subsequently, solid sarkosyl (*N*-lauroylsarcosine, sodium salt; Sigma-Aldrich) was added to the 1S supernatant to achieve a 1% concentration and stirred for 1 hour. Thereafter it was centrifuged at 100,000 *g* for 1.5 hours at RT. Following centrifugation, pellets were gently rinsed with 1 ml of the extraction buffer and centrifuged at 100,000 *g* for 20 minutes at RT. The pellets containing sarkosyl-insoluble tau fractions were dissolved in SDS-PAGE loading buffer to a final volume corresponding to the 1/50 of the volume of 1S supernatant used for their preparation. For Western blot analysis, 6 μl of the sarkosyl-insoluble fraction was loaded, which corresponds to 30 mg of tissue.

### Immunoblot analysis

Samples of sarkosyl-insoluble tau fractions [23] were dissolved in 1× SDS sample loading buffer in 1/50 volume of the soluble fraction and heated at 95°C for 5 minutes. Each sample in a quantity of 6 μl was then loaded onto 5–20% gradient SDS-polyacrylamide gels and electrophoresed in a Tris-glycine-SDS buffer system for 40 minutes at 25 mA. Proteins were transferred to PVDF membranes (1 hour at 150 mA in 10 mM *N*-cyclohexyl-3-aminopropanesulfonic acid, pH 12). After the transfer, the membranes were stained with Ponceau S (Additional file 1) for verification of loading of an equal amount of sarkosyl-insoluble proteins and efficiency of electroblotting. Subsequently, the membranes were blocked in 5% nonfat dry milk in PBS for 1 hour at RT and then incubated for 1 hour with primary (tau-specific) monoclonal antibodies, followed by three washes with a large volume of PBS. After washes, HRP-conjugated goat anti-mouse Ig (Dako) diluted 1:4,000 with PBS was used as a secondary antibody. Incubation (1 hour at RT) was followed by washing (three times) with 0.2% Igepal in PBS. The blots were developed with SuperSignal West Pico Chemiluminescent Substrate (Pierce Biotechnology), and the signal was detected using the LAS-3000 Imaging System (FUJI Photo Film Co, Tokyo, Japan). The signal intensities were quantified using AIDA Image Analyzer software (Raytest, Straubenhardt, Germany) and then statistically evaluated. Foetal tau (prepared as described in [31]) in the amount of 0.6 μg/lane was used as an internal standard for quantification.

### Immunohistochemistry of rat brain tissue

Transgenic rats were deeply anaesthetised with Zoletil-xylasine and perfused intracardially using a peristaltic pump for 2 minutes with PBS. The brain was frozen in liquid nitrogen and transferred to dry ice. Sagittal brainstem sections (10 μm thick) were cut on a Leica CM1850 cryomicrotome (Leica Biosystems, Buffalo Grove, IL, USA). The sections were postfixed with 4% paraformaldehyde (PFA) in PBS, pH 7.2 (4% PFA), for 10 minutes. Tissue sections were incubated with primary antibodies AT8 (Pierce Endogen), pT212 and pS214 (Invitrogen, Carlsbad, CA, USA), Tau5 (a gift from Dr Lester I Binder) and HT7 (Pierce/Thermo Scientific, Rockford, IL, USA) overnight at 4°C. Sections were immunostained using the standard avidin-biotin-peroxidase method (VECTASTAIN ABC Kit; Vector Laboratories, Burlingame, CA, USA) with VIP as chromogen.

### Immunohistochemistry of human brain tissue

Human postmortem AD brain tissue (Braak stage VI) was obtained from the Netherlands Brain Bank in accordance with local ethical approval and written consent from the donor or the donor’s next of kin. Ethical approval was obtained for the analyses carried out using this tissue (Institute of Neuroimmunology, Slovak Academy of Sciences, 5/2011).

Tissue sections from human AD brain were treated with cold (+4°C) 99% formic acid for 1 minute at RT (25°C). Brain sections were incubated for 20 minutes at RT in 0.01 M of PBS, pH 7.4, containing 0.3% Triton X-100 and 1% H_2_O_2_, followed by a 30-minute incubation in the blocking solution (0.01 M of PBS, containing 0.3% Triton X-100, 1% horse serum), followed by overnight incubation with sera from transgenic rats immunised with the tau peptide vaccine (diluted 1:1,000) at 4°C. After washing, the sections were immunostained using the standard avidin biotin peroxidase method (ABC Elite Kit; Vector Laboratories). The reaction product was visualised using Vector VIP as a chromogen (Vector Laboratories). Sections were then examined with an Olympus BX51 microscope.

### NeuroScale

For testing of sensorimotor functions a multi-test battery with a novel sensitive scoring system—NeuroScale [32]—was chosen. Briefly, NeuroScale consists of three variants of beam walking test (square cross-section of 3 cm × 3 cm, rectangular cross-section of 4 cm × 2 cm, round cross-section with diameter of 3.5 cm), a prehensile traction test and a rapid neuromuscular and neurological examination. Animal performance was evaluated and scored according to a predefined rating scale. The maximal number of points possible to achieve in the beam walking and prehensile traction tests was five each. Performance on a simple reflex response was scored with a maximum of one point, except the hindlimb escape extension reflex, for which we used a 3-point rating scale.

### Statistical analysis

Statistical analysis was carried out using the Prism statistical software package (GraphPad Software, La Jolla, CA, USA). To compare two groups, the Mann–Whitney *U* test or an unpaired *t*-test was applied. The results are presented as mean ± standard error of the mean unless otherwise specified. Differences were considered significant at the level of *P* < 0.05.

### Preclinical toxicology and safety pharmacology studies

Studies were conducted at good laboratory practice level at Harlan Laboratories SA (Correzzana, Italy). Four separate formulations of AADvac1 vaccine (10, 40 and 160 μg of peptide coupled to KLH formulated with aluminium hydroxide containing 1.5 mg of aluminium per dose and 40 μg of peptide coupled to KLH formulated with aluminium hydroxide containing 0.5 mg of aluminium per dose) were compared to placebo.

Single-dose studies consisted of three separate rat studies with 14 days or 21 days of postadministration daily clinical observation. Animals were weighed twice per week, and food consumption was recorded weekly. The 14-days studies featured complete haematology, urinalysis, coagulation and clinical biochemistry panels. Animals were killed on day 15, organ weights of all internal organs were measured and histopathological analysis of all tissues was performed. The 21-days study featured clinical observation after administration of four times the intended human clinical dose (160 μg).

Chronic toxicity testing was conducted in New Zealand white rabbits. Animals received 9 doses of AADvac1 or placebo in 3-week intervals over the course of 26 weeks. Animals were clinically observed twice daily. Food consumption and body weights were recorded weekly. The study featured complete haematology, urinalysis, coagulation and clinical biochemistry panels. Ophthalmoscopic examinations were performed at baseline and after 13 and 26 weeks. After the animals were killed, organ weights of all internal organs were measured and histopathological analysis of all tissues was performed.

An acute central nervous system (CNS) safety pharmacology study was conducted in ICR (CD-1) outbred mice according to a modified Irwin screen test paradigm. Animals were subjected to detailed neurological examination for 24 hours after drug administration. An acute cardiorespiratory safety pharmacology study was conducted in Beagle dogs. The cardiovascular (arterial pressure, heart rate, cardiac contractility (dP/dt_max_), systemic blood flow and electrocardiography) and respiratory parameters (rate, tidal volume and minute volume) were recorded and analysed before and 5, 15, 30, 45 and 60 minutes after placebo or vaccine administration. Histopathological analysis was peer-reviewed by AnaPath GmbH, Oberbuchsiten, Switzerland. Vaccine and placebo for all toxicology and safety pharmacology studies was provided by Bachem Distribution Services GmbH, Weil am Rhein, Germany.

## Results

### Active tau peptide vaccine containing structural determinant of tau protein essential for its pathological interaction generates antibodies discriminating between pathological and physiological tau

In a tandem paper, we report that the monoclonal antibody DC8E8 revealed structural determinants of the tau protein that are important for the pathological tau–tau interaction [30]. Thus, targeting these interaction domains could lead to the successful treatment of AD and related tauopathies. The identified tau determinants served as a lead for the design of an active peptide vaccine, which was used in therapeutic efficacy studies in a transgenic rat model of AD—SHR72 [25]. We prepared an active vaccine based on the cysteinated tau peptide C-^294^KDNIKHVPGGGS^305^, which contains the structural determinant ^299^HVPGGG^304^[30]. The peptide was conjugated via its N-terminus to a carrier (KLH) and formulated so that a single dose contained 100 μg of the conjugated peptide.

**Figure 1:**
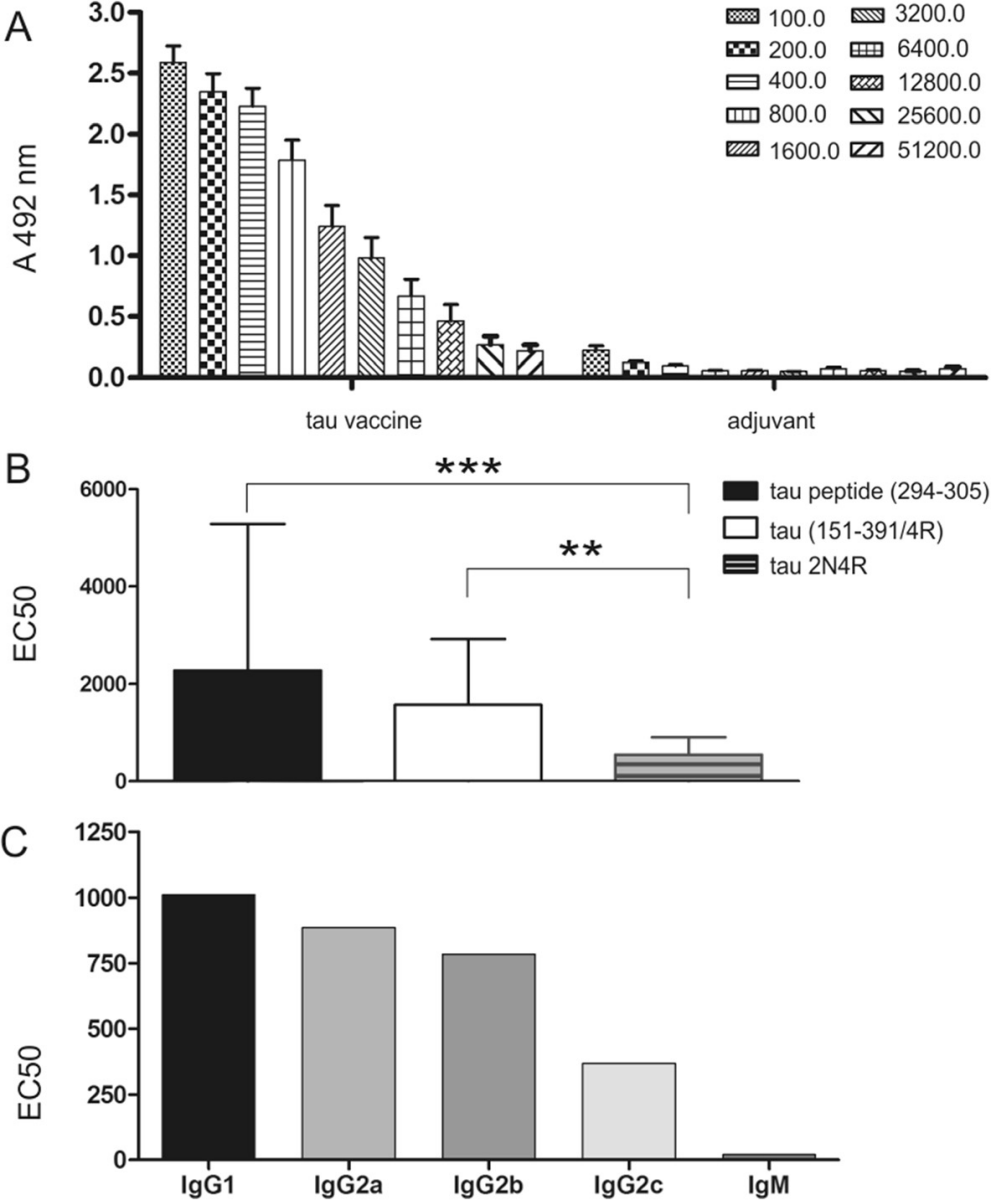
**Antibody response in transgenic rats immunised with tau peptide vaccine determined by enzyme-linked immunosorbent assay.** Tau peptide vaccine induces high antibody levels specific to the tau peptide ^294^KDNIKHVPGGGS^305^. No antibody response was observed in the mice immunised with adjuvant only. Data shown are serial twofold dilutions of animal sera **(A)**. Antibodies elicited by vaccination exhibited statistically significantly higher binding activity to tau peptide and to mis-disordered tau (151-391/4R) than to full-length tau 2N4R (***P = 0.0003 and **P = 0.0028, respectively) **(B)**. EC50, Half-maximal effective concentration. Vaccine-induced antibodies specific to mis-disordered tau are prevalently of the immunoglobulin G (IgG) isotype **(C)**. The data shown are means with error bars representing SD.

For this study, transgenic rats expressing human mis-disordered tau were chosen. We have previously shown that these transgenic rats developed extensive NFTs and axonal degeneration in the brain [25,33]. The neurodegeneration correlated well with neurobehavioural changes [32]. Transgenic rats were immunised with five doses of the vaccine, starting at 2 months of age, followed by the second injection 3 weeks later and thereafter on a monthly schedule. The experiment was concluded when the animals were 6.5 months of age. At this age, blood was collected from the heart. The sera were used for determination of antibody response to the vaccine. Serial dilutions (1:100 to 1:51,200) of each serum were tested against the synthetic peptide ^294^KDNIKHVPGGGS^305^, against mis-disordered tau (151-391/4R) and against physiological tau 2N4R using indirect ELISA. The tau peptide vaccine induced a robust antibody response in immunised rats (Figure 1A). No specific antibodies were observed in rats immunised with adjuvant only. The geometric mean titres (GMTs) of antibodies specific to the tau peptide (Figure 1A) and to mis-disordered tau reached high values (21,000 and 15,000, respectively). On the contrary, the GMT of antibodies recognising full-length tau 2N4R (4,000) was more than fivefold lower than that recognising the immunogen. Importantly, the analysed antibodies showed statistically significantly higher binding activity to the tau peptide and to mis-disordered tau 150-391/4R, than to physiological tau 2N4R (*P* = 0.0003 and *P* = 0.0028, respectively) (Figure 1B). Vaccination with tau vaccine carrying DC8E8 epitope induced antibodies preferentially recognising mis-disordered tau protein, thus discriminating between pathological and physiological tau.

### Antibodies specific to mis-disordered tau induced by vaccination are predominantly of immunoglobulin G isotypes

To determine the isotypes of the antibodies produced in response to the tau vaccine, sera from rats were serially diluted (1:100 to 1:12,800) and tested against mis-disordered tau (151-391/4R) using ELISA. Vaccination of rats with the tau peptide vaccine preferentially induced formation of IgG1 antibody isotypes specific to mis-disordered tau (151-391/4R) (Figure 1C), indicating a predominant type 2 helper T cell (Th2) type of immune response. Moreover, the presence of high levels of IgG1 antibodies with preferential affinity to mis-disordered tau points to the therapeutic potential of such antibodies. By contrast, the isotypic profile showed low levels of IgM antibodies, which are considered to have low affinity to the antigen.

### Vaccination significantly reduces tau oligomerisation and pathological tau phosphorylation

**Figure 2:**
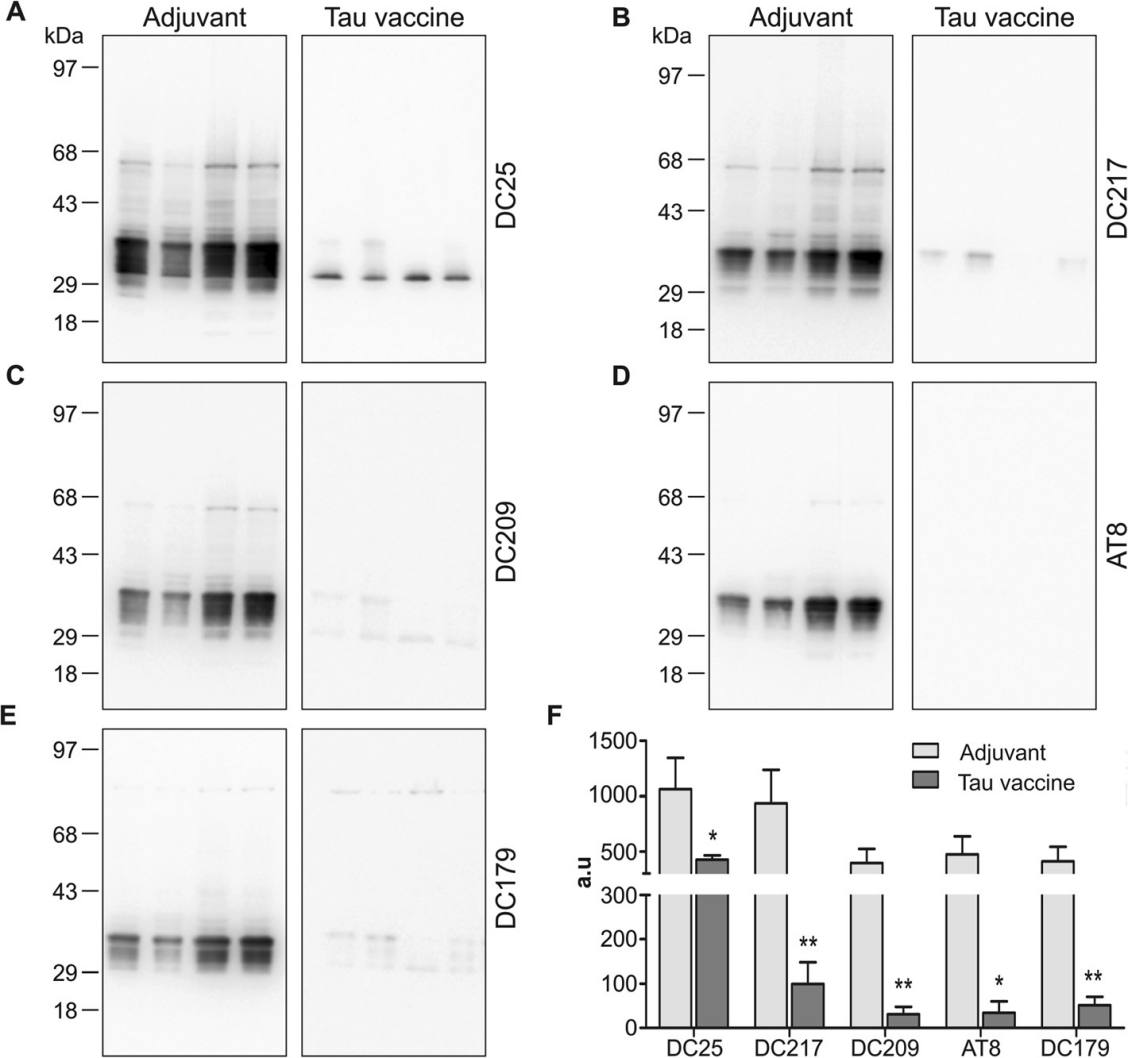
**Immunisation with tau peptide vaccine reduced tau oligomers and tau hyperphosphorylation.** Western blot analysis with pan-tau monoclonal antibody DC25 showed reduction in oligomeric tau in the brain of transgenic rats treated with tau peptide vaccine **(A)**. The monomeric endogenous rat tau proteins run between 43 and 68 kDa marker bands, whereas monomeric transgenic tau comprises multiple phospho-species between 29 and 43 kDa marker bands. In the vaccine-treated animals, there are only remnants of nonphosphorylated transgene running just above the 29 kDa marker band. Western blot analysis revealed significant reduction of hyperphosphorylated tau species phosphorylated at Thr217 (monoclonal antibody (mAb) DC217) **(B)**, pThr231 (mAb DC209) **(C)**, pSer202/pThr205 (mAb AT8) **(D)** and pThr181 (mAb DC179) **(E)**. **(F)** The graph represents the quantification and statistical evaluation of the difference between animals treated with vaccine and those treated with adjuvant only; *P < 0.05, **P < 0.01 . A 6-μl sarkosyl-insoluble fraction was loaded per lane, which corresponds to 30 mg of tissue. Loading of an equal amount of sarkosyl-insoluble proteins, and the efficiency of electroblotting was verified by staining the membrane with Ponceau S (Additional file 1).

We have previously shown that a transgenic rat model expressing misfolded truncated tau protein developed extensive neurofibrillary pathology. The proteomic analysis of NFTs revealed that insoluble tau consisted of monomeric truncated tau (about 30 kDa), highly phosphorylated forms of truncated tau (30 to 36 kDa), tau oligomers (above 36 kDa) and full-length tau (above 43 kDa) [25]. Because the AD pathology in transgenic rats is caused by pathological insoluble tau forms, the impact of treatment on the tau oligomers was analysed. The brainstems of the treated and untreated transgenic rats were used for the extraction of sarcosyl-insoluble pathological tau. Immunisation with tau vaccine induced significant reduction of the level of highly phosphorylated forms of truncated tau (30 to 36 kDa), tau oligomers (above 36 kDa) and full-length tau (above 43 kDa) revealed by measurement with pan-tau monoclonal antibody DC25 (*P* = 0.0322) (Figure 2A and F). Moreover, analysis with phospho-dependent monoclonal antibodies revealed significant reduction of the levels of insoluble tau species phosphorylated at Thr217 (pThr217, *P* = 0.0094; Figure 2B and F), pThr231 (DC209, *P* = 0.0077; Figure 2C and F), pSer202/pThr205 (AT8, *P* = 0.0112; Figure 2D and F) and pThr181 (DC179, *P* = 0.0100; Figure 2E and F). These results show that the immunisation significantly reduced early forms of pathological tau (represented by monomers, dimers and oligomers) and late forms of pathological tau polymers (represented by paired helical filaments (PHFs)) as well.

### Active vaccination decreased the number of transgenic rats with fully developed neurofibrillary pathology

**Figure 3:**
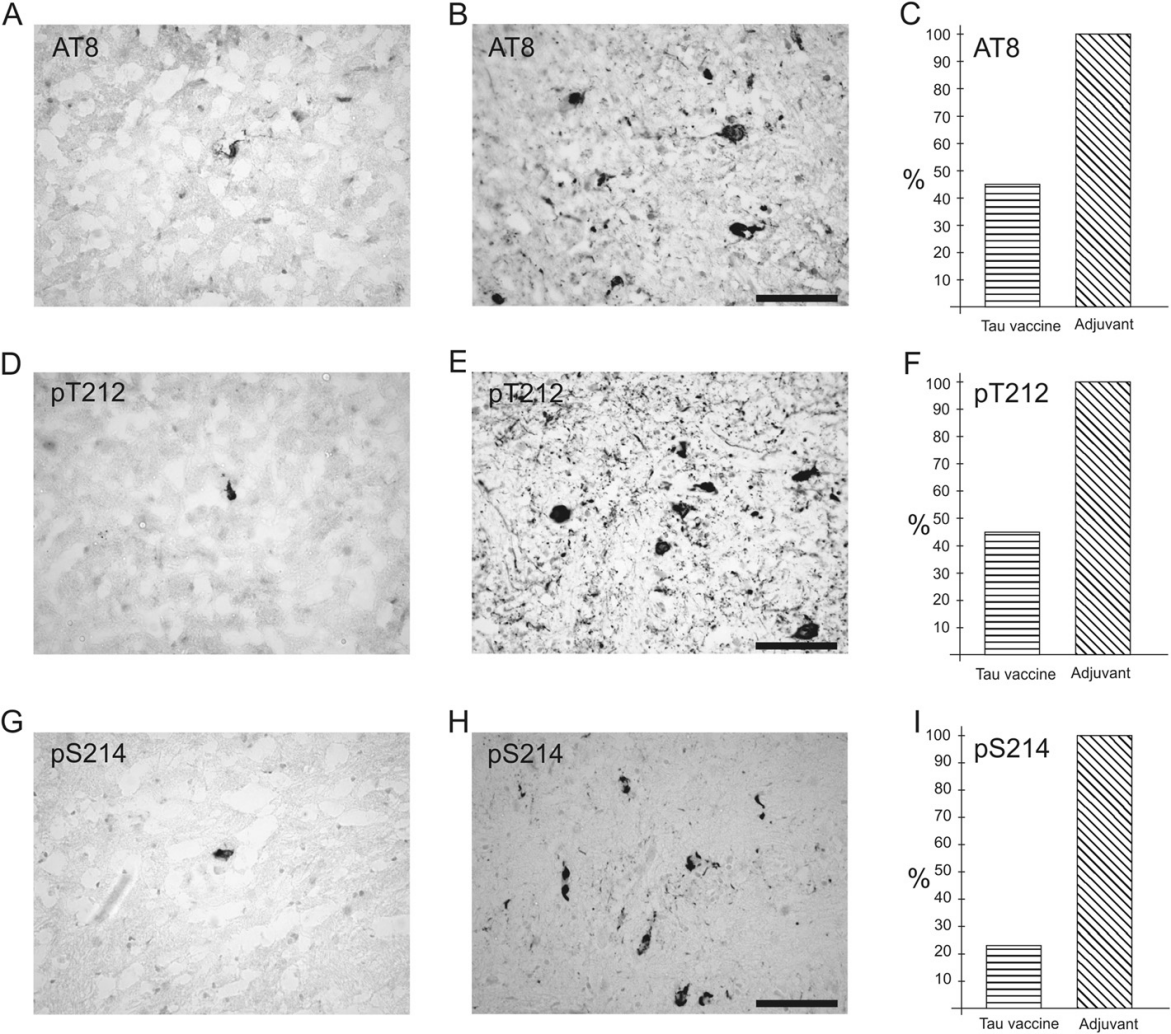
**Active vaccination reduced the number of transgenic rats developing extensive neurofibrillary pathology.** Immunostaining with AT8, pT212 and pS214 shows low numbers of neurofibrillary tangles in the brainstem of treated transgenic rats **(A)**, **(D)** and **(G)** compared with untreated transgenic rats **(B)**, **(E)** and **(H)**. Immunisation lowered the number of transgenic rats with extensive neurofibrillary degeneration by 55% **(C)** and **(F)** or by 77% **(I)**.

For determination of the therapeutic efficacy of the tau peptide vaccine, we used immunohistochemical methods to stain NFTs in serial cryosections of rat brains. We found that several monoclonal antibodies, including TAU5 (part A in Additional file 2), HT7 (B in Additional file 2), pT212 (C in Additional file 2) and pS214 (D in Additional file 2), recognised neurofibrillary degeneration in transgenic rat brain. For further quantification, we selected antibodies recognising pathological tau phosphorylation, such as pT212 and pS214, and antibody AT8, which is recommended as a marker for tau pathology in human AD [34]. We compared number of transgenic rats with extensive neurofibrillary pathology in the brainstem. The majority of treated transgenic rats displayed reduced numbers of NFTs (nNFTs; AT8, nNFTs <5; pT212, nNFTs <10; pS214 nNFTs <10; Figure 3A, D and G), whereas the majority of untreated transgenic rats showed higher numbers of NFTs (Figure 3B, E and H). Immunostaining with AT8, pT212 and pS214 showed that immunisation reduced the number of transgenic rats with extensive neurofibrillary degeneration by 55% (AT8, pT212; Figure 3C and F) or by 77% (pS214; Figure 3I).

### Active immunisation improves the sensorimotor functions of transgenic rats

**Figure 4:**
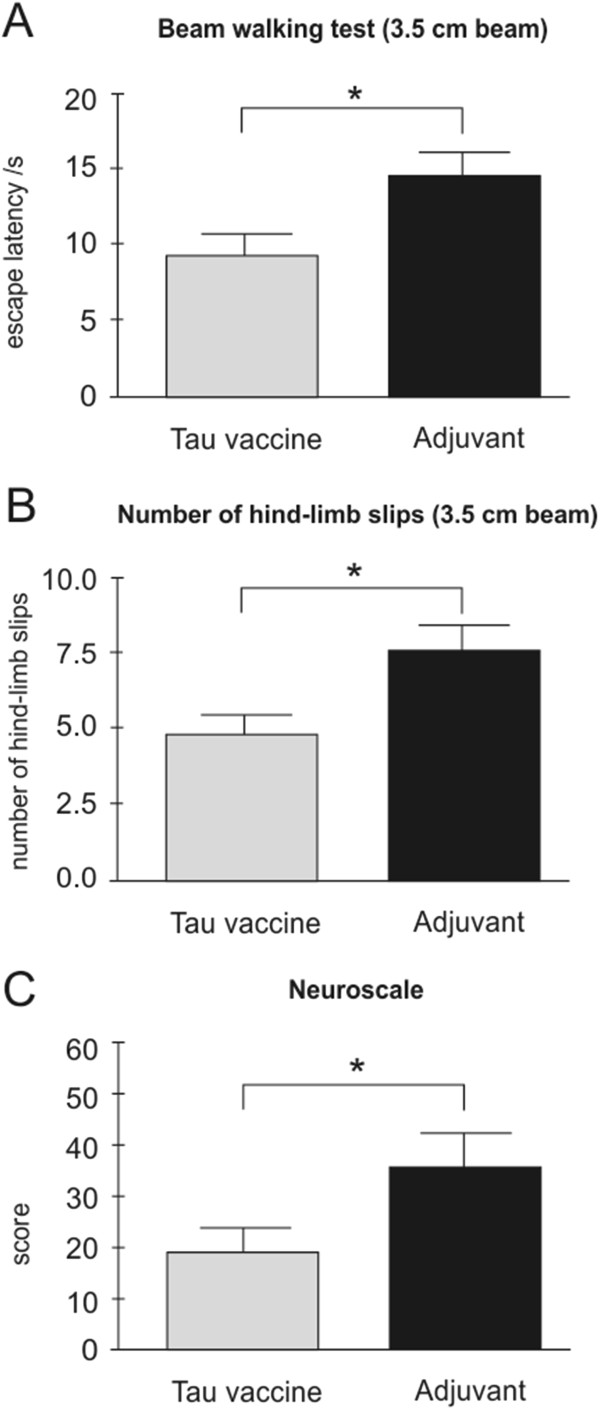
**Tau vaccine improves sensorimotor functions of transgenic rats.** Tau vaccine–treated transgenic rats showed decreased escape latency **(A)** and reduced hindlimb slips **(B)** compared to adjuvant-treated controls. Tau vaccine–treated transgenic rats had significantly lower NeuroScale scores than adjuvant controls, which reflects an improvement of sensorimotor functions **(C)**, *P < 0.05.

In order to test the improvement of sensorimotor functions of immunised transgenic rats, we used a battery of behavioural tests—NeuroScale [32]. At 6.5 months of age, transgenic rats treated with tau vaccine were subjected to behavioural tests with the aim of determining the effect of immunotherapy. Rats treated with the tau vaccine showed significantly decreased escape latencies (*P* = 0.04, Figure 4A) and reduced number of hindlimb slips (*P* = 0.045, Figure 4B) in the beam walking test compared to the transgenic rats that received adjuvant alone. The immunisation significantly improved the total NeuroScale score of the rats treated with tau vaccine compared to the control treatment group (*P* = 0.047, Figure 4C).

### Vaccination generated rat antibodies that recognised neurofibrillary degeneration in the Alzheimer’s disease brain

**Figure 5:**
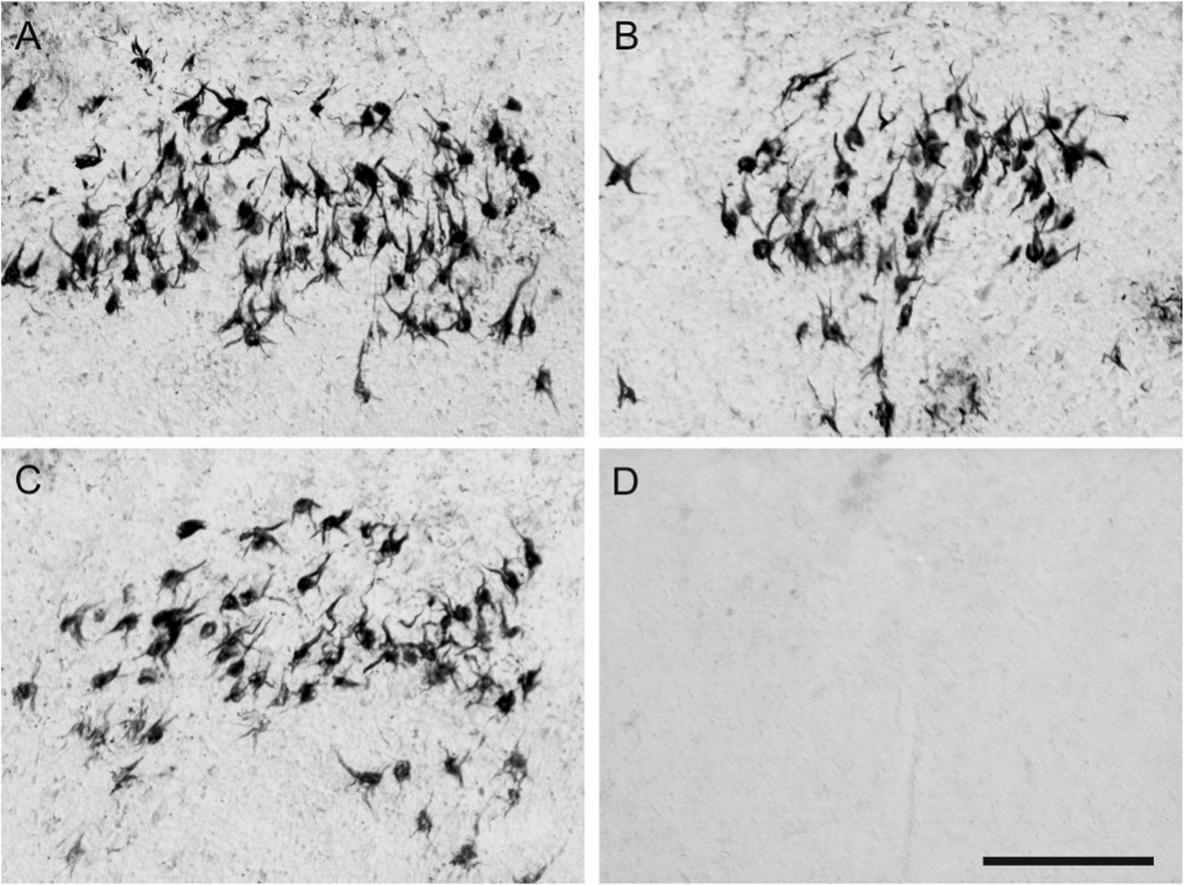
**Sera from transgenic rats immunised with tau peptide recognised neurofibrillary degeneration in the Alzheimer’s disease brain.** Three representative sera obtained from rats immunised with tau peptide recognised neurofibrillary tangles and neuropil threads in the entorhinal cortex of Alzheimer’s disease brain **(A)**-through **(C)**. In contrast, serum from a rat immunised with adjuvant did not recognise any neurofibrillary pathology **(D)**. Scale bar = 100 μm.

In order to validate the specificity of antibodies generated by active immunisation, we tested their ability to immunolabel neurofibrillary lesions in human AD brain. Three representative sera obtained from rats immunised with tau peptide were able to recognise NFTs and neuropil threads in the entorhinal cortex of AD brains (Figure 5A through 5C). Serum from a rat immunised with adjuvant only did not recognise any neurofibrillary pathology (Figure 5D).

### Active tau peptide vaccine displayed a favourable safety and tolerability profile in all toxicology and safety pharmacology studies

The vaccine was well-tolerated at all administered dose levels in all tested species throughout the course of all studies. No effects of toxicological relevance were detected in clinical observation, haematology, blood biochemistry, coagulation analysis, urinalysis, necropsy, histopathological analysis, CNS safety pharmacology or cardiorespiratory safety pharmacology. Measured by body weight gain, all study groups thrived equally. As expected of a vaccine containing aluminium hydroxide as adjuvant, subcutaneous administration induced reversible nodules up to 1.5 cm in their largest dimension at the injection site. The morphological study of the injection sites showed focal/multifocal subcutaneous granulomatous inflammation with abundant histiocytes consistent with the histological presentation of an aluminium granuloma.

To assess possible negative impacts on the immune system, thorough haematological analysis was conducted in acute and chronic toxicology studies in rats and rabbits, respectively. The haematology panel featured the following assessments: erythrocyte count, haemoglobin, haematocrit, mean corpuscular volume, red-cell volume distribution width, mean corpuscular haemoglobin, mean corpuscular haemoglobin concentration, haemoglobin concentration distribution width, reticulocyte count, reticulocyte maturity index, platelet (thrombocyte) count, total leukocyte count, differential leukocyte count (neutrophils, lymphocytes, monocytes, eosinophils, basophils, large unstained cells). Both absolute numbers and relative amounts of white blood cells were assessed. The vaccination did not induce any toxicologically relevant effect on any of the observed parameters.

## Discussion

The neurofibrillary pathology composed of disease-modified tau protein is the main pathological correlate for cognitive decline and memory impairment in AD and related tauopathies [1,3,35]. Thus, a variety of therapeutic approaches targeting the tau misfolding cascade have been proposed [11,36,37]. Tau immunotherapy represents an emerging disease-modifying strategy that can prevent or reduce tau oligomers and/or neurofibrillary pathology. Several independent studies showed that either active or passive immunotherapy can reduce tau hyperphosphorylation, prevent tau aggregation or clear tau oligomers and insoluble aggregates [13-16,18,19]. However, until now, no anti-tau immunotherapy has been reported to enter clinical trials in AD. In this study, we present preclinical efficacy results of the tau peptide vaccine, which is the prototype for the first-in-man tau vaccine that entered phase I of clinical trials in June 2013 under the name AADvac1 (details can be found on the Clinicaltrials.gov registry homepage under identifiers NCT01850238 and NCT02031198) with the aim of protecting tau from its pathological tau–tau interactions.

Previously, we identified the vulnerable, immunologically distinct structural determinants on pathological tau that are essential for its misfolding and oligomerisation [30]. With this knowledge, we developed a tau peptide vaccine encompassing the structural determinant derived from the mis-disordered second microtubule-binding repeat domain of tau. With the aim of improving immune response to the vaccine, the cysteinated 12-mer tau peptide was conjugated to the carrier protein (KLH). Previous active immunisation trials with a β-amyloid vaccine (AN-1792 [38,39]) showed severe adverse effects induced by a cell-mediated Th1 immune response, highlighting the importance of safety aspects of vaccine design. In our tau peptide vaccine, the helper T-cell epitopes are derived exclusively from the carrier protein due to the small size of the adopted tau peptide. Moreover, the vaccine is formulated with an adjuvant that promotes Th2 immune response [40]. In summary, the final vaccine formulation contains a B-cell epitope derived from tau and helper T-cell epitopes derived from KLH in combination with a Th2-promoting adjuvant, thus inducing a Th2 humoral antibody response directed against mis-disordered tau.

The tau vaccine elicited a robust and specific antibody response. Applied vaccine induced generation of high-affinity antibodies targeting pathological tau in immunised animals. Moreover, the immune response to the active vaccine was shifted towards the Th2 phenotype, underlining its safety. Taken together, our results demonstrate a highly favourable safety profile of the presented tau vaccine, which was confirmed by detailed toxicology studies.

An extensive review of the literature on the correlation of AD neuropathological changes with cognitive impairment clearly showed that the severity of cognitive impairment correlates best with the burden of neocortical NFTs [3]. Moreover, the number of NFTs correlates with disease duration, supporting the idea of a progressive accumulation of tau pathology. A very recent study revealed that NFT correlated with disease duration in both typical and atypical cases of AD, suggesting that distribution of tau pathology in different brain areas determines the specific clinical picture of dementia [2]. Furthermore, it has been shown that tau pathology is frequently seen in subcortical nuclei [41] and that the motor signs predict cognitive and functional decline, institutionalisation and mortality in AD [42].

We have previously shown that tau pathology is distributed in several brain areas of tau transgenic rats, including forebrain and subcortical areas similar to human AD [23-25]. Similar to AD, the distribution of tau pathology in the brain of transgenic rats determines the clinical outcome. We have demonstrated that transgenic rats have neurobehavioural impairment as measured by a battery of tests [32,43]. Interestingly, the active immunisation of transgenic rats with the tau peptide vaccine was shown to have a therapeutic effect on several levels. First, the vaccine displayed a disease-modifying effect on disease progression by targeting the main pathological AD hallmarks—tau oligomers—that correlate well with the clinical outcome. It reduced early (oligomers) and late (PHFs) manifestations of tau pathology and thus improved the health status of transgenic rats. Second, immunisation led to a significant decrease of tau phosphorylation on several AD-related phospho-sites, such as pThr181, Ser202, pThr205, pThr217 and pThr231. It is important to note that the majority of other preclinical tau vaccine studies have shown reduction of tau phosphorylation in transgenic mouse brains, mostly on those phospho-sites that were part of the original immunogen [13,18,19]. In contrast to those studies, our tau immunotherapy attacks very early stages of tau oligomerisation that precedes tau hyperphosphorylation, thus removing the mis-disordered tau substrate for hyperphosphorylation. This is in line with the notion that tau therapy should not target just one or two phospho-sites, because of the pronounced heterogeneity of phospho-tau species in the AD brain that participate in tau oligomerisation.

It has been shown in studies on transgenic mice that tau active immunisation can improve various neurobehavioural parameters in treated animals. Tau vaccination increased the time the mice were able to stay on the accelerating rotarod and reduced the number of foot slips in the traverse beam task [13] or improved short-term memory in the Y maze [19]. In our study, we tested the efficacy of the tau peptide vaccine using the NeuroScale—a battery of behavioural tests featuring a novel scoring system for the phenotyping of the transgenic rat model of tauopathy [32]. Our results clearly show that vaccination improved the sensorimotor impairment of transgenic rats.

Finally, it is important to note that vaccination elicited specific rat antibodies that were able to recognise tau lesions, including NFTs and neuropil threads in the AD brain. Therefore, it is reasonable to expect that the elicited antibodies will exert the same pattern of therapeutic activity in AD patients.

## Conclusions

In the present study, we have thoroughly characterised a new type of active tau vaccine. On the transgenic rats we demonstrate that the tau vaccine is (1) immunogenic—induces a strong antibody response, (2) specific—targets only one antigenic site on tau, (3) selective—discriminates between pathological and physiological tau, (4) safe—helper T-cell epitopes are derived exclusively from the carrier protein (KLH), and the immune response is shifted to the Th2 phenotype, (5) therapeutically effective—reduces pathological tau phosphorylation and pathological tau oligomers and (6) improves neurobehavioural parameters. It is important to highlight that the active tau peptide vaccine AADvac1, based on the tau peptide ^294^KDNIKHVPGGGS^305^, after successfully passing through toxicology and safety pharmacology studies, has already entered the phase I clinical trial phase.

## Abbreviations

AD: Alzheimer’s disease; Aβ: Amyloid-β peptide; CNS: Central nervous system; ELISA: Enzyme-linked immunosorbent assay; GMT: Geometric mean titre; GMBS: *N*-[γ-maleimidobutyryloxy]succinimide ester; HRP: Horseradish peroxidase; KLH: Keyhole limpet haemocyanin; NFT: Neurofibrillary tangle; PBS: Phosphate-buffered saline; PHF: Paired helical filament.

## Competing interests

EK, NZ, BK, PN and MN are employees of AXON Neuroscience SE and do not own any shares of the company. The authors declare that they have no other competing interests.

## Author’s contributions

EK developed the active vaccine and performed immunological analyses, including interpretation of data. NZ performed histological analyses, including interpretation of data. BK was instrumental in the neuroproteomic part of the study and formulation development of the vaccine, including interpretation of data. PN was instrumental in the preclinical toxicology and safety pharmacology studies and formulation development of the vaccine, including interpretation of data. MN designed the immunogen of the vaccine, conceived of and designed the study and drafted the manuscript. All authors read and approved the final manuscript.

## Supplementary Material

Additional file 1 The Ponceau S staining of representative membranes after Western blot transfer of sarkosyl insoluble tau. Click here for file

Additional file 2 Immunostaining of the transgenic rat brain demonstrates that NFTs are recognized by different monoclonal antibodies including TAU5, HT7, pT212 and pS214. Click here for file
