# Supplementary material for: First-in-man tau vaccine targeting structural determinants essential for pathological tau–tau interaction reduces tau oligomerisation and neurofibrillary degeneration in an Alzheimer’s disease model
Source: Alzheimers Res Ther. 2014 Aug 1;6(4):44. doi: 10.1186/alzrt278 (PMC4255368; doi:10.1186/alzrt278)
Supplement: Additional file 1 — The Ponceau S staining of representative membranes after Western blot transfer of sarkosyl insoluble tau. [file alzrt278-S1.docx]

Additional file 1

The Ponceau S staining of representative membranes after Western-blot transfer of sarkosyl insoluble tau.


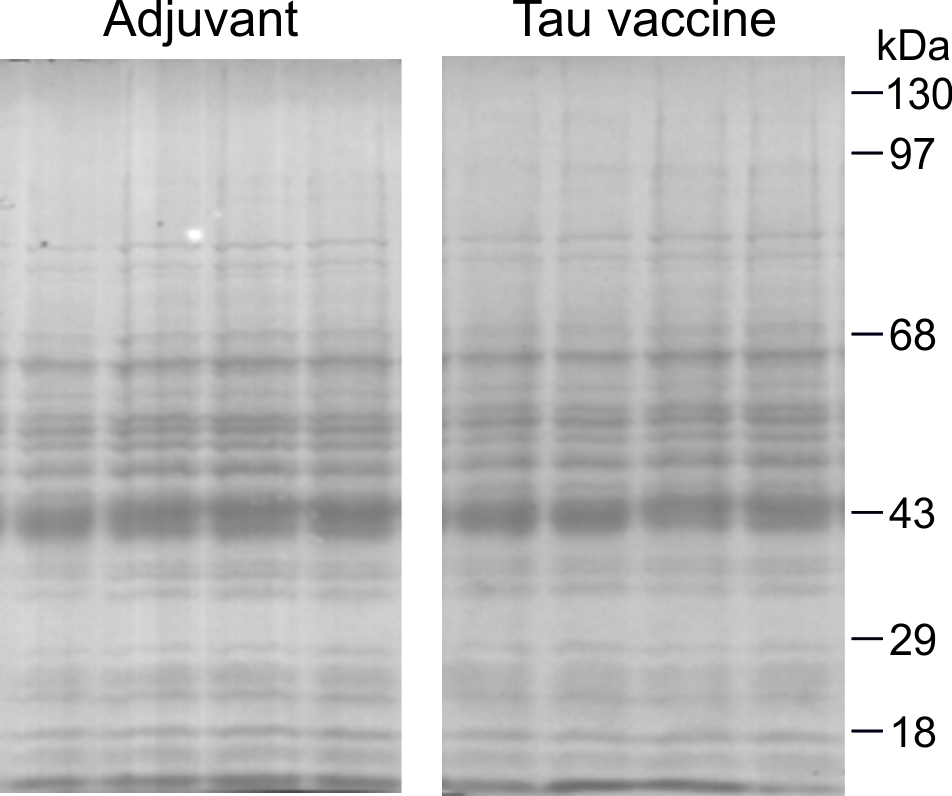
6 µl of the sarkosyl insoluble fraction was loaded per lane, which corresponds to 30 mg of tissue. The membrane was stained with Ponceau S. As judged from the staining intensities, all lanes contained comparable amount of proteinaceous material.
