# Supplementary material for: First-in-man tau vaccine targeting structural determinants essential for pathological tau–tau interaction reduces tau oligomerisation and neurofibrillary degeneration in an Alzheimer’s disease model
Source: Alzheimers Res Ther. 2014 Aug 1;6(4):44. doi: 10.1186/alzrt278 (PMC4255368; doi:10.1186/alzrt278)
Supplement: Additional file 2 — Immunostaining of the transgenic rat brain demonstrates that NFTs are recognized by different monoclonal antibodies including TAU5, HT7, pT212 and pS214. [file alzrt278-S2.docx]

**Additional file 2**

Immunostaining of the transgenic rat brain demonstrates that NFTs are recognized by different monoclonal antibodies including TAU5 (A), HT7 (B), pT212 (C) and pS214 (D). For the quantification we have selected pT212 and pS214 antibodies and AT8 as a widely used marker for tau pathology. Scale bar = 50µm.


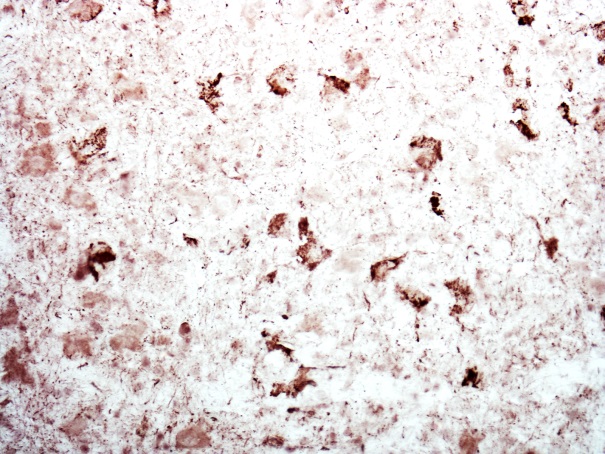

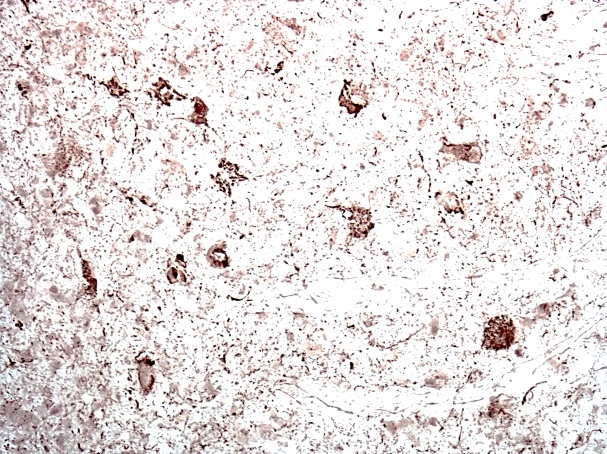


**B**

**A**


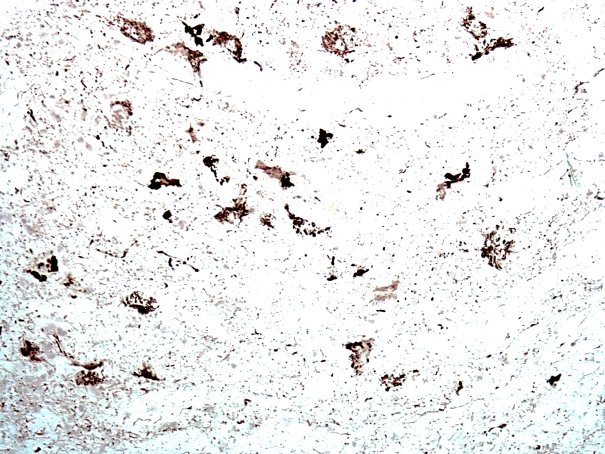

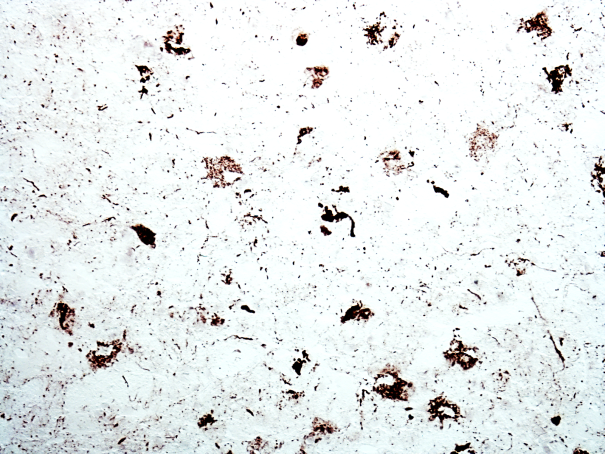


**D**

**C**
